# Mega infrastructure projects and their contribution to sustainable development: the case of the Athens Metro

**DOI:** 10.1007/s10644-023-09493-w

**Published:** 2023-03-15

**Authors:** Roido Mitoula, Angelos Papavasileiou

**Affiliations:** Department of Economics and Sustainable Development, School of Environment, Geography and Applied Economics, Harokopio University of Athens, Athens, Greece

**Keywords:** Mega infrastructure projects, Sustainable development, Social sustainability, Three pillars of sustainable development, Public transport, Athens Metro, Greece, Q01, Q50, Q56, R11, R40, R42

## Abstract

This paper examines the critical role of Mega Infrastructure Projects in sustainable urban and peripheral development by presenting a Sustainable Infrastructure Serum Analysis supported by primary field research. In the Athens Metro case study, we examined the project's impact on sustainable development by analysing the opinions of the project's users. As a result, the Athens Metro serves as a case study to help us better understand sustainable infrastructure as a framework for green growth from the standpoint of society. The three pillars of sustainable development are inextricably linked. This study focuses on the social comprehension and acceptance of a Mega Infrastructure Project effects. We attempt to demonstrate the interdependence of the three pillars of sustainable development through public opinion responses to our research by developing a statistical model fed by public responses to a prototype questionnaire that we developed to support our research objectives. The study's findings highlighted the project's social acceptability and necessity by establishing a direct positive correlation between sustainability, society, the economy, and the environment from the standpoint of society.

## Introduction

Transport is a critical infrastructure project and necessary in modern urban environments (Skayannis and Kaparos 2013). The aim is to meet the daily needs of the population and product movements (Mitoula et al. 2008). Today, citizens’ quality of life depends on an efficient and accessible transport system. However, at the same time, transport can be detrimental to the environment and contribute to climate change (Halkos et al. 2020). Studies have shown that in the European Union, the transport sector consumes one-third of the total final energy, mainly from oil, and that transport contributes to a quarter of the total greenhouse gas emissions (Mitoula and Economou 2008). While most other economic sectors, such as electricity generation and industry, have reduced their emissions since 1990, transport emissions have increased. Cars, vans, trucks, and buses produce more than 70% of greenhouse gas emissions from transportation. (Halkos et al. 2014) It is noted that the remaining emissions come mainly from shipping and air transport (New Climate Economy 2016). In addition to the above issues concerning the effects of transport on climate, there are other adverse effects: transport infrastructure occupies large areas of land, supports urban sprawl, and divides natural areas into smaller sections, with severe consequences for animals and plants.

Nevertheless, urban rail networks and the Metro have grown rapidly around the world and play an essential role in the growth of cities (Ward and Skayannis 2019). Their significance stems from the numerous benefits they represent. Combined with the rapid growth of urban centres and overcrowding (Alaimo et al. 2022), these benefits have made urban railway networks and the Metro essential structural and functional elements of a modern city (Mitoula et al. 2003). The present work refers to the Athens Metro, a mega infrastructure project in the transport field (Skayannis 2021). The influence of the project on sustainable development is investigated through the opinion of its users based on the model of the fundamental pillars of sustainable development (Vardopoulos 2021). Thus, the Athens Metro provides a case study to improve our understanding of the concept of Sustainable Infrastructure as a framework for Green Growth through social opinion as the users and city experience the influence of Athens Metro in their daily life. The study’s goal is to compile data on the Athens Metro and its synergy with the city’s and country’s sustainable development so that its multifaceted impact on the locals and visitors to Athens, as well as the economy and market at large, can be better understood.

Furthermore, the findings from the questionnaire collection contain sufficient and valuable information about the operation, the pros and cons of the Athens Metro, and its reputation among the citizens who use it and benefit from it in various ways. Finally, the research implications can contribute to the design of future studies for the Athens Metro, aiming to evaluate the contribution of large construction projects to sustainable development. Sustainable development’s economic, social, and environmental pillars are undeniably interdependent (Vardopoulos et al. 2020). The social pillar is a crucial and necessary component of the success of the fundamental model of three pillars, encompassing social well-being, societal and economic growth, and security. This investigation will focus primarily on social comprehension and acceptance of the Athens Metro’s effects on sustainable development. We attempt to confirm the interdependence of the three pillars model for sustainable development through public opinion and responses to our research. In comprehending the society impacted by this mega infrastructure project, we attempt to determine whether or not the economy, society, and environment are basic foundational elements of sustainable development. To support our research objectives, we developed a statistical model of the interdependence formulas of the three pillars of sustainable development, complete with dependent and independent variables, which be fed by public responses to a prototype questionnaire designed to evaluate and measure the public understanding and acceptance of mega infrastructure projects' contribution to sustainable development.

## Literature Review

### The three pillars of sustainable development

In the past two decades, there has been an explosion in the number of publications on the topic of “sustainability,” to the point where “sustainability science” is now commonly regarded as a separate subject. Despite this, “sustainability” remains a vague notion that can be understood in various ways depending on the surrounding circumstances (Purvis et al. 2018). A common way to explain the concept of “Sustainable Development” is by referring to the economy, society, and environment as three separate but interdependent “pillars”. These “pillars” can be represented graphically as supporting sustainability or intersecting circles. As Purvis, Mao and Robinson 2019 highlighted, the lack of literature conceptualising “sustainability” and “sustainable development,” the “three pillars” of environmental, economic, and social, have gained support. This is usually done by balancing seemingly equally desirable goals within these three categories but uses vary. One problem with this conceptualisation is its lack of theoretical development; it seems to arise in the literature and be taken at face value.

Nevertheless, this method has been presented as a shared vision of sustainable development since 2001 (Giddings et al. 2002). Geiger et al. 2021 research used the three-pillar sustainability model as a conceptual framework to examine how individuals evaluate climate policies and how these evaluations predict policy support. We consider individuals’ evaluations of 1) environmental impacts (i.e. perceived policy effectiveness), 2) economic impacts emerging, and (3) social impacts of policies. Sustainable development has implications for major project planning, evaluation, and implementation (Vardopoulos et al. 2021). Environmental and social elements have a significant impact on project planning and delivery. The concept of sustainability is still in its infancy and its operationalisation. It is uncommon for all environmental, social, and economic factors to be addressed within a project (Dimitriou, Harman and Ward, 2010).

### Social sustainability

The social component is one of sustainability’s foundations. However, social components are evaluated less frequently than economic and environmental dimensions. Infrastructure development includes the planning, construction, operation, and eventual decommissioning of a service or facility to meet a public need. In this regard, infrastructures serve as an intermediary link that enables sustainable social development (Tsilika and Vardopoulos 2022). The project and society may be harmed if the social dimension is neglected during infrastructure development. Non-reversal impacts that may jeopardise intergenerational life quality have long-term effects on future generations’ development (Sierra et al. 2018). As Sierra et al. 2017 mentioned in the research of methods for estimating the social sustainability of infrastructure projects, according to Colantonio (2011), social sustainability can be defined as both a situation and a process that works to enhance the quality of life in a community. According to Asomani-Boateng (2015), one of the characteristics of social progress is the improvement of interventions. Others connect ecological and economic viability with the health of the present and future generations (Valdes-Vasquez and Klotz, 2013; Mostafa & El-Gohary 2014). The ways in which infrastructure is conceived, constructed, put to use, and eventually abandoned will define the social repercussions of the infrastructure (Sierra et al. 2016). According to Valdes-Vasquez and Klotz (2013), it is essential for the design and planning of an infrastructure project to consider the context of the region, the user, the commitment of relevant stakeholders and the identification of those stakeholders (Anastasiadou et al. 2022). A significant number of societal repercussions are contingent on either pre-existing conditions or immediate actions (Van de Walle 2009). In several contexts, socio-economic development in the short term can be detrimental to underdeveloped regions (Foth et al. 2013). Consequently, it is important to ensure that distribution systems include those most susceptible to harm (Mostafa & El-Gohary 2014). People are interested in what will happen in the long run. Using goals and criteria for social development might help you figure out whether or not a project’s infrastructure will be able to support itself over time (Pavlovskaia 2013). The majority of the societal expectations of the 1990s were satisfied by Labuschange et al., 2005. Focusing on long-term social reform goals in a zone is more vital. The types of social indicators are utilised to decide the path that must be taken to improve society (Fulford et al. 2015). A social indicator is a tool that may be used to evaluate how well a society is achieving its development goals or bettering its citizens’ lives over time (Noll 2013). When it comes to determining the social sustainability of infrastructure, the knowledge gap presents itself in two different ways: (1) the social contribution of infrastructure in terms of how it interacts with its surroundings (Gannon & Liu 1997; Van de Walle 2009; Asomani-Boateng et al. 2015); and (2) the possible long-term benefit distribution effects balanced against its short-term contribution. (1) The social contribution of infrastructure in terms of how it interacts with its surroundings (Colantonio 2011; Foth et al. 2013; Sierra et al. 2016). These are the foundational pieces upon which this inquiry is built.

### Quality of life and sustainable infrastructure

The provided quality of life improvement is a foundational piece of sustainable development that describes another aspect of sustainable infrastructure growth (Cortesi et al. 2022). In 1995 Manfred Max-Neef defined the “threshold hypothesis”, stating that ‘for every society, there seems to be a period in which economic growth brings about an improvement in the quality of life but only up to a point—the threshold point—beyond which, if there is more economic growth quality of life may begin to deteriorate. Dimitriou, Harman & Ward, 2010 state that equal access to services promotes individual and communal quality of life. These issues receive little attention because they are frequently political; they are more difficult to characterise since measurement often necessitates judgement. Social factors must alleviate poverty. This is essential to Brundtland’s sustainable development strategy and a World Bank aim. The environment and society are inextricably interwoven (Zopounidis et al. 2020). Disasters and failures worldwide show the need to preserve ecosystems for human economic and social well-being (Skayannis and Zafeiriou 2021). Any infrastructure project involves environmental and social risks. Comprehensive project assessments should identify and weigh all critical factors, but they are never certain. There may be environmental and social risks. For example, the volume of complaints about an infrastructure project’s environmental impact can force rethinking and rerouting a project section, increasing development and construction costs and incurring delays.

Furthermore, problematic transportation access to a major city reduces local centre activity, employment opportunities, and the accessibility of town centre facilities to poor communities, raising the project’s cost for public sponsors. Some project influence factors are difficult to predict and may represent minor or major risks; however, if they enter, the consequences might be severe (Dimitriou, Harman and Ward, 2010). Failure to appropriately examine environmental and social factors can result in substantial risks, such as losing the support of key stakeholders, failing to identify how to fulfil stakeholder objectives, or causing unacceptable consequences that are too expensive to repair (Passas et al. 2022). As part of their infrastructure development plans, more project sponsors emphasise developing and presenting a “sustainable business case.” However, a project’s environmental, social, and economic factors are rarely balanced. Decision-making often necessitates trade-offs to attain project goals and objectives (Vardopoulos 2019). To handle the risks, uncertainties, and tensions associated with these trade-offs, suitable and transparent institutional capacity and governance frameworks are required. This is critical since many institutional frameworks for major projects are too fragmented and isolated to allow for satisfactory compromises. Few infrastructure stakeholders now publicly believe economic growth should be the sole or significant consideration in project evaluation. According to Dimitriou, Harman and Ward, 2010, 81% of survey respondents related to the infrastructure industry; economic development should not be the only criterion. Sustainability is still perceived in multiple ways (for example, see; Zorpas 2014; Egidi et al. 2020; Kavouras et al. 2022; Ragazou et al. 2022). Therefore, to achieve successful sustainable development, societal acceptance and enhancing the quality of life of the users and communities engaged in the infrastructure project should be addressed (Fischer and Anekudzi 2011).

### Transportation projects importance to emerging economies

The need for transportation services, and by extension, energy, will continue to be driven by the rise of the population. On the other hand, the shift toward urbanisation will give rise to very different cities. Although it is anticipated that the world's largest cities will have more than 10 million residents by 2030, many of the world's cities with the highest population growth are smaller settlements with fewer than 500,000 people. (Tsafos 2019) Because of its larger population and higher population density, a city with more than 10 million residents will have distinct energy issues than a metropolis with 500,000 or 1 million inhabitants (Halkos and Tzeremes 2014). Therefore, along with population expansion and urbanisation, the industry will be a major driver for transportation services. Transport services form a network linking various nodes in industrial supply chains. Moving people and things from one location to another entails transportation, which also involves a variety of other considerations such as cost, convenience, time, and safety. (Tsafos 2019) Full-time jobs transporting people and products and delivering urban services like food are made possible by ride-sharing services in emerging economies, making them a potential route out of poverty. When exactly oil demand will peak is a topic of heated discussion. (Halkos and Tzeremes 2011) Even though the EIA predicts demand will keep rising until 2040, other analysts predict it will decrease much sooner. Several areas, like sustainable development and economic growth in the transportation sector, require policy guidance, and discussions on this topic must be broadened (Tsafos 2019). The provision of transportation services must include safety features for the public. Data are essential for discovering novel patterns, yet it is often inaccessible or behind expensive paywalls. To properly control data, governments must adopt a methodical and comprehensive strategy. Using social and behavioural policy, governments should "promote" public transportation as the better choice. Fuels are becoming available to power motorbikes, automobiles, trucks, trains, and eventually ships and aeroplanes, causing urban areas to evolve and new business models to emerge (Halkos and Tzeremes 2011) (Tsafos 2019).

### Literature Summary and Research Gap

Considerable literature indicates a positive link between mega infrastructure project investments and sustainable development (indicatively; Mitoula and Patargias 2002; Mitoula et al. 2013; Vardopoulos and Theodoropoulou 2018; Mitoula and Papavasileiou 2021; Papavasileiou and Mitoula, 2021). However, some researchers cite the possible negative impacts of such projects, socially, on social and environmental developments. As such, controversy exists in this field of study. Besides, while some previous studies offered key insight into the socio-economic impact of megaprojects and the concept of sustainability, little research exists into the impact of mega infrastructure projects investments on sustainable development under the prism and consideration of society. On the other hand, some of the studies offered valuable insights by considering the challenges in assessing megaprojects’ impacts on socio-economic and environmental development but not under the measurable opinion of the users and communities affected by this mega infrastructure project. This presents a research gap the proposed study deems worthy of further exploration.

## The case study of a mega infrastructure project, the “Athens Metro.”

**Fig. 1:**
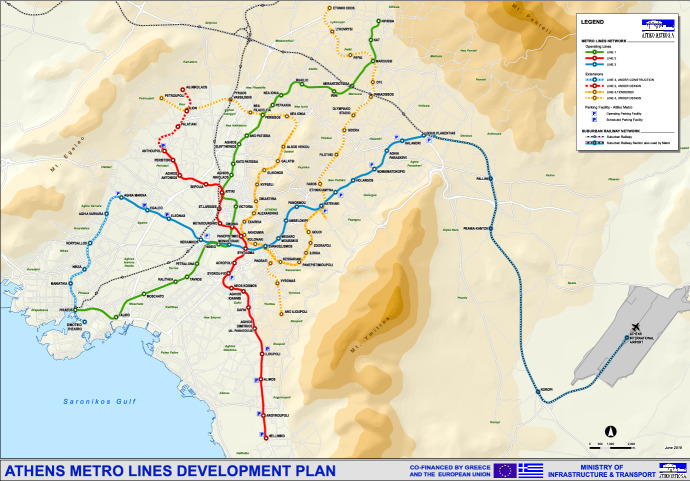
Athens Metro Lines Development Plan Map, Source: https://www.ametro.gr/wp-content/uploads/2018/06/AM_Sxedio_Anaptiksis_Jun18_en.pdf

The Athens Metro consists of 3 lines: (1) Line 1, which is the pre-existing electric railway that has been operating since 1869 initially as a steam train (Attiko Metro SA 2021c), (2) line 2, which extends from Elliniko to Anthoupolis, and 3) line 3, extending from Nikea to Doukissa Plakentias (see map Fig. 1). The Basic Project of the Athens Metro began construction in November 1992 with a planned 20 km network with 21 stations on 2 Lines. The first 13 km with 14 stations in Line 3 and Line 2 were put into operation in January 2000, while 5 additional km with 5 stations were in operation in November 2000. Given the existence of important antiquities in Athens, the construction company ATTIKO METRO SA funded archaeological excavations of 69,000 square metres, which are the largest ever made in the area. In addition, to minimise the chances of encountering archaeological finds, the Metro tunnels were drilled, on average, to a depth of more than 15 m, a level lower than where archaeological finds are usually found. In April 2003, the Syntagma—Monastiraki section was opened to the general public. In June 2004, the section Dafni—Agios Dimitrios, 1.2 km, was put into operation, followed by, in July 2004, the sections Ethniki Amyna-Chalandri, D. Plakentias, with a total length of 5.9 km. In August of the same year, the extension Sepolia—Agios Antonios began operation with a total distance of 1.4 km. In 2013, another 7 stations with a total length of 8.5 km were delivered to the general public. On July 6, 2020, the first 3 stations to Piraeus were opened to passengers for use: Agia Varvara, Korydallos and Nikaia. It is noted that Attiko Metro SA. has designed the stations that were put into operation with an emphasis on bioclimatic characteristics and the safe movement of passengers in the Metro network. With the operation of the first three stations of the extension to Piraeus, the additional passengers total on the network is estimated at 63,000 per day. At the same time, the residents of the Municipalities of Agia Varvara, Korydallos and Nikaia have a modern Metro line at their disposal. The operation of the first three stations two years earlier than the completion of the full extension of Line 3 of the Metro has significant benefits, socio-economic and others, such as positive effects in tackling climate change: reducing car traffic vehicles by 11,000 per day and carbon dioxide emissions by 60 tonnes per day. The entire Line 3 Expansion is expected to be completed in the summer of 2022.

It is important to emphasise that with the completion of the project, a significant Transport Centre is planned to be created at Piraeus Station, operationally joining two Metro lines (Lines 1 and 3), the Port, the Suburban Railway, and the Tram Extension to Piraeus (5,4 km single line and 12 stations), thus facilitating transfers between all modes of transport. In addition, the connection between the Port of Piraeus and the Airport “Eleftherios Venizelos” through Metro Line 3 will provide unique development benefits to the greater area of Athens and Piraeus well to the national economy in general. The Metro is the most important means of transportation in Athens and extends to 59.7 km, and 938,000 passengers are served daily by 43 modern stations (Attiko Metro SA 2021a) (Table 1).

**Table 1:** Attiko Metro Lines-lengths-stations -daily ridership 2021

Metro lines in operation	Length (kilometres)	Stations	Daily ridership
Line 1 (ISAP)	25,6	24	460.000 passengers
Line 2 and 3 (Metro)	59,7	43	938.000 passengers
Total	**85,3**	**67**	**1.398.000 passengers**

Highlighted results that are significant in the analysis and discussion indicated in bold

*Data: Attiko Metro S.A*

The operation of the Metro is carried out electrically. Most of its route is underground with an exclusive corridor in conventional or fixed structures (Patargias et al., 2004). Regarding the environmental impact of the Metro in the city, its contribution is considered significant. The improvement of public transport has reduced the use of private vehicles in the centre of Athens, thereby improving the quality of the environment and the standard of living of the city’s residents. According to a recent study for future extension of line 4A, the amount of CO2 would be reduced by 38 percent by 2030 compared to the current situation.

Furthermore, Attiko Metro S.A. claims that the extension of Metro line 3 to Piraeus will have significant socio-economic and other benefits, such as a reduction in car traffic vehicles by 11,000 per day and a reduction in carbon dioxide emissions by 60 tonnes per day. Furthermore, related to the completion of the project and the operation of the stations: Maniatika, Piraeus, and Municipal Theatre, it is anticipated that total passenger traffic in the Metro network will increase by 132,000 citizens per day, reducing private vehicle traffic by 23,000 per day and carbon dioxide emissions by 120 tonnes per day (Attiko Metro SA 2021b). One of the primary drivers behind the construction of the Athens Metro was the desire to alleviate traffic congestion in the city centre and, as a result, improve environmental conditions (Batsos and Tzouvadakis 2007). The radio-centric development of the Athenian urban structure has caused several problems, particularly in densely populated areas. Traffic on the roads is a significant issue, particularly during peak hours, when the travel time to a location by car can be tripled compared to the rest of the day. As a result, in addition to contributing to reducing environmental problems, another reason for the Metro’s creation was to reduce road traffic (Dimitriou et al. 2014). We can see this from the growing number of people who use the Metro daily. In particular, if this population travels by Metro, traffic on Athens’ streets is reduced by approximately 938,000 cars per day (Attiko Metro SA 2021a). Resolving this major issue elevates the Metro project to the top of Athens’ priority list. It should also be noted that it allows people to travel who do not have the financial means to purchase a private vehicle.

## Research methodology

According to Dwigo and Dwigo-Barosz 2018, the primary function of a research methodology is to define the process or methods utilised to collect data. The applied methodology begins with extensive bibliographic analysis, followed by parallel evaluations of more classic and current research approaches. Next, a prototype primary questionnaire survey was conducted, supplemented by collecting secondary data about infrastructure project information. Finally, the data were analysed statistically through STATA software based on several criteria.

### Research objective

The primary objective of this research is to investigate the role of the Athens Metro mega infrastructure project in sustainable development by analysing society’s perspective on the project’s impact on the three dimensions of sustainable development: the economy, the environment, and society.

### Research method

Primary data were collected using prototype survey questionnaires administered to select adult participants. The questionnaire addresses the impact of infrastructure on the environment, the economy, and social life. As a result, the questions are tailored to the environmental, social, and economic impact of mega infrastructure in sustainable development. Thus, the primary questions for the survey reflect the social opinion of the effects of the Athens Metro mega infrastructure project by the interrelation of sustainable development fundamental pillars. The questionary target participants include literate adults with adequate knowledge about infrastructure and its implications on the economy, the environment, and people’s lives. Permanent residents of Attica’s region, urban, and suburban settings were prioritised because they have adequate experience in their respective areas crossed or served by the Athens Metro. As a result, they are more likely to observe this mega infrastructure project’s effects on all aspects of their lives and surroundings. In addition, individuals of any employment or job status were eligible to participate in the research, provided they were permanent residents of their respective areas. For instance, a permanent resident will more likely recognise the primary purpose of infrastructure, and infrastructural implications on the people, the economy, and the environment.

#### Research desίgn

A questionnaire containing mostly dichotomous, ranking, and multiple-choice questions was used for quantitative research, with the respondents’ degree of agreement measured. Because the concepts of sustainable development and user satisfaction with the infrastructure project are measurable, this type of questionnaire is considered appropriate (Creswell, 2013). Furthermore, quantitative research has the advantage of storing large data. As a result, a large sample size can be used, as in the current study. In addition, the quantitative approach is the suitable research style for the present study when correlation analysis is necessary, according to the research questions. Correlations are effectively investigated in quantitative research since mathematical and statistical approaches are applied (Muijs, 2010).

#### Research tools

In our research, we used the following research tools:

##### Demographic questionnaire

- 8 closed-ended questions and short answers

##### Research questionnaire

- 12 questions, dichotomous type (Yes / No)
- 4 Ranking question
- 3 Multiple choice questions

#### Research sample

Consequently, a questionnaire-based survey was conducted from October to December 2020 to meet the demands of the research case study. The sample consisted of 266 Attica Region residents. Because of the COVID-19 pandemic’s restrictions on movement, surveys were issued and collected online. The study findings were organised and processed using Microsoft Forms, open-source software. The STATA software was used to analyse the data. The questionnaire includes questions about the Athens Metro’s influence on the environment, the economy, and social life. The research was carried out by sending and completing 27-question surveys through email and social media. Facebook and email were the most often utilised modes of communication.

#### Main research questions

*The Five (5) main research questions* related to sustainable development that were selected to be analysed and correlated with the demographic characteristics of the respondents with statistical analysis are the following:

- Sustainability
  **Q9**. *Did the surrounding Prefectures/Municipalities Areas develop or generally benefit from the operation and construction of the infrastructure project?*
  The question will guide data collection on the benefits of infrastructure for authorities within the project site. The question seeks to understand the benefits of major infrastructure projects, including Sustainable Development.
- Society
  **Q10**. *In your opinion, has residents’ quality of life in the project area improved?*
  The question assesses the impact of infrastructure on the overall quality of life. The main aspects include job creation, promoting social opportunities and providing services to people.
- Environment
  **Q12**. *Did the infrastructure project contribute positively to the environmental impact of the surrounding areas?*
  The statement will serve as a determining factor in the impact that infrastructure has had on the environment. The expected responses to the statement included yes or no, which will help determine the project’s value in promoting environmental Sustainability.
- Economy
  **Q20**. *In your opinion, was there an increase in trade in the broader project areas?*
  The increase in trade determines the importance and influence of the project in the region’s economic development with direct results on Society. Thus, enhancing business through the presence of an infrastructure project establishes economic and social prosperity and contributes to the people’s overall well-being. Therefore, this answer will help determine the value of infrastructure for economic development.
- User Satisfaction
  **Q27.** *Are you satisfied with the quality of the infrastructure project?*
  The level of satisfaction experienced by end users is inextricably linked to the degree to which society acknowledges the practicability and long-term viability of the mega infrastructure project. Through the use of this question, we are attempting to get unambiguous evidence that the infrastructure project’s end users are pleased.

#### Factor reliability

Table 2 presents the results of the reliability analysis for the factors. It turns out that for the Sustainable Development Pillars model, it is *α* = 0.742, and for User Satisfaction, it is *α* = 0.875.

**Table 2:** Reliability analysis

Factors	Questions	Cronbach Alpha
Sustainable Development Pillars model	9, 10, 12, 13	0.742
User Satisfaction	9, 27	0.875

#### Ethical considerations

Before accessing infrastructure and relevant authorities, we asked permission, including the local government and stakeholders. All the participants consented before participating in the research. No participant was coerced to participate in the study to ensure willing participation and openness to responding to the survey. In addition, the anonymity of the participants was maintained by concealing their names to uphold privacy and confidentiality. As a result, no autobiographical information was collected. Further, the research is not used to implicate property owners and major stakeholders responsible for constructing major infrastructure. All the guidelines are documented and attached to the research questionnaire before data collection.

## Empirical analysis

### System of equations

**Fig. 2:**
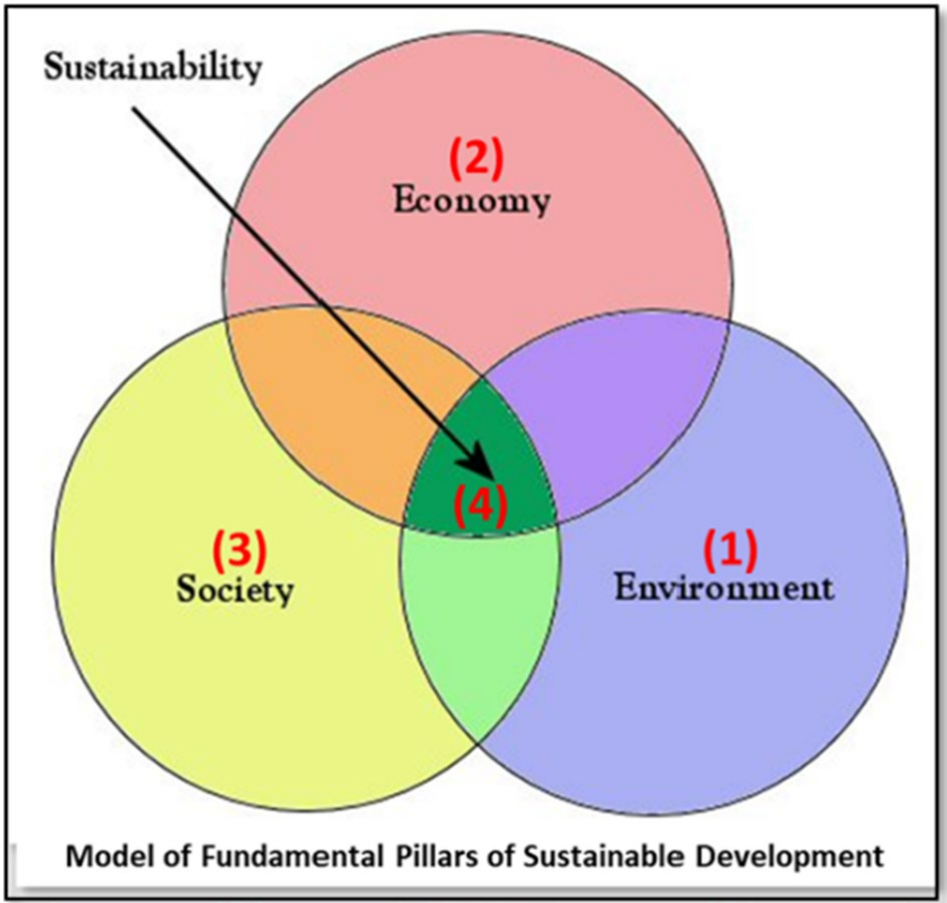
Interaction between the three fundamental pillars of Sustainable Development in Mega Infrastructure Projects

The interrelationship between the three pillars of sustainable development represented by the variables (Table 3) of environment, quality of life and commerce and their effect on sustainable development/growth (Fig. 2) can be expressed mathematically as a system of equations:

1 $${\text{Env}} = \beta_{0} + \beta_{1} {\text{Qual}} + \beta_{2} {\text{Comm}} + \varvec{X\theta } + \varvec{Y\rho } + u$$

2 $${\text{Qual}} = \gamma_{0} + \gamma_{1} {\text{Env}} + \gamma_{2} {\text{Comm}} + \varvec{X\mu } + \varvec{Z\pi } + \eta$$

3 $$Comm = \delta_{0} + \delta_{1} Env + \delta_{2} Qual + \varvec{X\zeta } + \varvec{W\tau } + \varphi$$

4 $$Growth = \alpha_{0} + \alpha_{1} Env + \alpha_{2} Qual + \alpha_{3} Comm + \varvec{X\rho } + \varvec{V\sigma } + \psi$$

**Table 3:** Basic model variables definitions

Variable name	Definition
area_growth	*Q9—Did the surrounding Prefectures/Municipalities Areas develop or generally benefit from the operation and construction of the project?*
commerce	*Q20—In your opinion, was there an increase in trade in the wider area of the project?*
qual_life	*Q10—Has residents' quality of life in the area surrounding the project improved?*
environment	*Q12—Did the infrastructure project contribute positively to the environmental impact of the surrounding areas?*
satisf	*Q27—Are you satisfied with the quality of the infrastructure project?*

The three pillars of growth (environment, quality of life and commerce) are denoted by ${\text{Env}}$, ${\text{Qual}}$, and ${\text{Comm}}$, respectively. In addition, all equations include a vector of explanatory variables ${\varvec{X}}$ that is common across all equations. This contains controls for various demographic and socio-economic characteristics of the respondents. Specifically, all equations include controls for gender, age, education, marital status, urban areas, and employment status of the respondents. Furthermore, each equation includes in its specification a set of variables that is unique for the specific equation and excluded from the other equations, denoted by ${\varvec{Y}}$, ${\varvec{Z}}$, ${\varvec{W}}$, and ${\varvec{V}}$. This helps with the identification of the above system of equations and the estimation of the parameters of interest.

Although each Eq. (1)-(4) could be estimated independently, this would neglect the interrelationship between the three pillars of growth. Therefore, in order to capture this interdependency, we estimate Eqs. (1)-(4) as a system of equations, where the four key variables of interest are simultaneously determined within the system while also allowing for correlation between the error terms ($u$, $\eta$, $\varphi$, and $\psi$).

All four outcome variables in Eqs. (1)-(4) are binary, taking the value of one of the individuals who reported an improvement about the particular outcome and zero otherwise. The equations are estimated using a Linear Probability Model (LPM). 1The LPM is simply the application of Ordinary Least Squares (OLS), where the dependent variable is a binary variable instead of a continuous variable. The major advantage of LPM is its interpretability, as the estimated coefficients refer to changes in the probability that the outcome variable takes the value of one.

### User satisfaction

After studying people’s perceptions in relation to the three pillars of growth and growth itself, we focus on what makes people more receptive to infrastructure projects by examining users’ overall level of satisfaction with such projects.

The user satisfaction equation is given by:

5 $${\text{Satisf}} = \lambda_{0} + \lambda_{1} {\text{Growth}} + \varvec{X\xi } + \varvec{Q\kappa } + \nu$$

where ${\text{Satisf}}$ is a binary variable (Table 3) taking the value of one if the respondent overall is satisfied with the infrastructure project and zero otherwise. ${\varvec{X}}$ is the same vector as the one used in the analysis above, containing demographic and socio-economic variables and controls for the type of project. ${\varvec{Q}}$ is another vector containing other determinants of user satisfaction. Finally, $\nu$ is the error term.

## Statistical Analysis Results

### Discussion

#### Empirical findings

Following a careful examination and analysis of the descriptive statistics, correlations, and statistical regression analysis results, it is clear that the quality of life (qual life) defined as the pillar of Society in the theoretical model of sustainable development positively correlates with sustainable development. In addition, the results of the descriptive statistics in Table 4 give high acceptance rates to all the questions about the positive influence of the Athens Metro project that we set as fundamental pillars of the statistical model of sustainable development. The Quality of Life Improvement question (qual_life) recorded 90.2%, the Commerce Development question (commerce) 93.6%, the Environmental Impact question (enviroment) 75.9% and the project’s positive contribution to the development of the project areas. (area_growth) 92.9%, Table (4).

**Table 4:** Research questionnaire descriptive statistics

Descriptive statistics	Percent
***Q9. Did the surrounding Prefectures/Municipalities/Regions develop or generally benefit from the construction and existence of the infrastructure project?***	
***Yes***	**92.86**
*No*	7.14
***Q10. Has residents' quality of life in the area surrounding the project improved?***	
***Yes***	**90.23**
*No*	9.77
Q11. In order of importance, rank the following using reasons for infrastructure project use: *(most important first and least important last)*	
Commute to work	
***1***	
***Transportation (Products/Goods)***	
***4***	
***Travel (In remote areas of the city)***	
***3***	
Entertainment (Night Out, Social Activities, Shopping)	
***2***	
***Q12. Did the infrastructure project contribute positively to the environmental impact of the surrounding areas?***	
***Yes***	**75.94**
*No*	24.06
Q13. In your opinion, was the increase in jobs due to the existing infrastructure project in the surrounding areas greater, equal, or less compared to the reduction that may have been caused?	
***Greater***	**68.05**
*Equal*	21.81
*Less*	10.15
Q14. Can you report damages caused to the surrounding areas that the infrastructure project crosses?	
*Financial*	13.53
***Environmental***	**34.96**
*Social*	9.02
***None***	**42.48**
Q15. Can you rank the damages caused by the project in order of importance (minor, major, nil)?	
***Financial***	
*minor*	
***Environmental***	
*minor*	
***Social***	
*Nil*	
Q16. How have the prices of land use in the surrounding areas been affected?	
***Increased***	**91.35**
*Decreased*	8.65
Q17. Has land use changed in the surrounding areas of the project?	
*Yes*	**58.65**
*No*	3.76
*I do not know*	37.59
Q18. Which land use types are developed mostly in the areas the infrastructure project crosses?	
*Industrial District*	13.16
***Permanent Residences***	**53.01**
*Holiday Homes*	0.00
*Tourist facilities*	4.51
*Wholesale Trade*	2.63
*Open spaces—Urban & Suburban green*	3.01
*Urban centres*	6.02
*Merchandise Centre*	10.53
*Public Utilities Infrastructures*	1.13
*Urban Infrastructures*	2.26
*Other*	3.76
Q19. Which activities in the surrounding area were affected by this project, whether they increased, remained unchanged, or decreased?	
***Hotels***	
*increased*	
***Restaurants***	
*increased*	
***Coffee shops***	
*increased*	
Museums	
*increased*	
***Cultural / Historical sites***	
*increased*	
***Commercial shops***	
*increased*	
Industries	
*unchanged*	
***Agricultural activities***	
*unchanged*	
***Q20. In your opinion, was there an increase in trade in the wider area of the project?***	
***Yes***	**93.26**
*No*	6.37
Q21. What reasons, in your opinion, make this project an important infrastructure and investment for the region? Rank the reasons in order of importance. *(first most important and last least important)*	
***Development***	
***1***	
Financial	
***5***	
Trading	
***4***	
Social	
***3***	
***Usability***	
***2***	
Q22. In your opinion, what else could be done to improve the project’s functionality?	
*Different design*	28.95
***Capacity/size***	**53.38**
*Cost of use*	17.67
Q23. Are the costs of using the infrastructure project preventing it from being used?	
*Yes*	29.32
***No***	**70.68**
Q24. Are the prices for using the project reasonable and affordable for people who use it frequently?	
***Yes***	**68.05**
*No*	31.96
Q25. Is the project maintained, in your opinion, with diligence?	
Yes	70.68
*No*	29.32
Q26. Do you think there are construction or design failures in the project that expose its users to danger?	
***Yes***	31.95
*No*	**68.05**
***Q27. Are you satisfied with the quality of the infrastructure project?***	
***Yes***	**84.96**
*No*	15.04

The estimated parameters of Eqs. (1–4) are presented in Table 7. The reported coefficients refer to changes in the probability that individuals responded positively about the specific outcome. For example, in the first equation on the perceived quality of life, the coefficients refer to the change in the probability that an individual believes that the quality of life improved when the respective explanatory variable increases by one unit. The test confirms that the residuals from the four equations are correlated, thus, supporting our choice of estimation strategy.

The interdependence between the three pillars of growth is confirmed across all corresponding estimates. Specifically, examining people’s perceptions about whether a particular infrastructure project improved the quality of life (Eq. 2), we see that people who think that the project had a positive impact on the environment are also more likely to report that the quality of life improved, with a probability of reporting improvement in the quality of life higher by 31.9 percentage points. Similarly, there is a positive association between perceived benefits in commerce and improvements in quality of life. People who see commercial benefits from an infrastructure project are estimated to have a higher probability, by almost 38 percentage points of reporting that the quality of life improved as well. Both estimated effects are statistically significant at the 1% level.

In addition, the quality of life is found to be significant in all model equations in the regression, which is important because it is the focus of the research that analyse public opinion and understanding of the contribution of infrastructure projects toward the acceptance of sustainable development and its positive effects on people’s daily lives. Therefore, those who see an improvement in their quality of life (Social pillar of the Sustainable development model) due to the infrastructure project may also be more likely to see improvements in the surrounding environment (Environment Pillar), commerce (Economy pillar), and the growth (Sustainable Development). Because of this, the regression coefficients for quality of life in the model’s equations are 69 percentage points at the environment equation, 27 percentage points for commerce, and 21 percentage points for growth.

This positive and statistically significant correlation (Table 5) between the three pillars of sustainable development  is also confirmed in the estimates presented in Table 7. Overall, the findings support our initial hypothesis on how interrelated the three pillars of sustainable development are. Focusing on the magnitude of the estimated effects, we can also make some remarks about the relative importance and level of interdependence between the three pillars. For example, people’s perceptions of the environmental impact of an infrastructure project seem to matter, but commerce seems to matter less compared to the other two pillars. On the other hand, people’s perceptions of whether the quality of life has improved are found to be more important in explaining either of the other two pillars.

**Table 5:** Correlations -Pillars of Sustainable Development

	*area_growth*	*commerce*	*qual_life*	*environment*
*area_growth*	1.0000			
*commerce*	0.0469	1.0000		
*qual_life*	0.2528*	0.2245*	1.0000	
*environment*	0.0829	0.1046	0.2590*	1.000

Asterisk (*) denotes statistical significance at 5%

Although female individuals, compared to males, are more likely to see improvements in quality of life from infrastructure projects. However, they seem to be more concerned about the impact of such projects on the environment. Similarly, middle-aged respondents (age40_50) are more likely to reply positively to the influence of the infrastructure project on the environment. Interestingly, socio-economic characteristics such as the education and employment status of the individuals do not seem to have affected people’s perceptions of how such infrastructure projects may impact the quality of life, the environment and commerce.

Turning our attention now to the determinants of growth (Eq. 4), evidence supports the hypothesis that individuals’ perceived improvements in quality of life, the environment and commerce are positively related with favourable views on the impact such infrastructure projects have on growth in the surrounding areas. Specifically, each pillar is estimated to have a positive and statistically significant effect on perceived growth. People who regarded that the quality of life improved as a result of infrastructure projects are more likely to consider that such projects enhanced growth in the surrounding area, with the corresponding probability for a positive response increasing by 21.3 percentage points. The perceptions of environmental and commerce impact seem to matter less in people’s assessment of whether there has been growth in the surrounding area.

The analysis thus far from Tables 6 and 7 confirms our priors that perceptions on the three pillars of growth, quality of life, the environment and commerce, are positively related to each other, and they all positively affect people’s assessment of growth in the surrounding area. However, the analysis presented results focused on the correlation and interrelationship between the three pillars and their effect on growth.

**Table 6:** Regression analysis

Seemingly unrelated regression						
Equation	Obs	Parms	RMSE	“R-sq”	chi2	P
qual_life	266	13	.2786898	0.1193	133.91	0.0000
environment	266	11	.405998	0.0978	93.44	0.0000
commerce	266	32	.2217602	0.1780	87.95	0.0000
area_growth	266	14	.2425586	0.1130	33.87	0.0022

Here we estimate a system of equations where we allow the error terms to be correlated. The estimates are based on linear probability models, with heteroskedasticity robust standard errors

**Table 7:** Regression analysis with the parameters of Equations Model (1,2,3,4)

Robust						
	Coef	Std. Err	z	P >|z|	[95% Conf.Interval]	
*qual_life (Eq. ***2****)**						
*Environment (environment)*	0.319***	0.044	**7.250**	**0.000**	0.233	0.405
*Commerce (commerce)*	0.379***	0.104	**3.650**	**0.000**	0.175	0.582
*Female (Q1)*	0.051	0.042	1.220	0.221	− 0.031	0.132
*age29_39 (Q2)*	0.050	0.052	0.950	0.341	− 0.052	0.152
*age40_50 (Q2)*	− 0.140	0.092	− 1.520	0.129	− 0.320	0.041
*age50plus (Q2)*	− 0.070	0.085	− 0.820	0.412	− 0.236	0.096
*University (Q3)*	− 0.010	0.039	− 0.250	0.803	− 0.086	0.066
*non_single (Q4)*	0.063	0.070	0.900	0.367	− 0.074	0.201
*non_urban (Q5)*	− 0.134*	0.074	**− 1.810**	**0.071**	− 0.279	0.011
*Working (Q7)*	− 0.073*	0.039	**− 1.850**	**0.064**	− 0.150	0.004
*Social_Damages (Q15)*	− 0.042	0.039	− 1.080	0.282	− 0.119	0.035
*price_prohibit (Q23)*	− 0.014	0.032	− 0.450	0.655	− 0.077	0.048
*New jobs (Q13)*	0.060	0.038	1.550	0.121	− 0.016	0.135
*_cons*	0.291***	0.109	**2.660**	**0.008**	0.076	0.505
*Environment (Eq. **1**)*						
*qual_life (qual_life)*	0.693***	0.087	**8.000**	**0.000**	0.523	0.862
*Commerce (commerce)*	0.005	0.131	0.040	0.970	− 0.251	0.261
*Female (Q1)*	− 0.094*	0.056	**− 1.670**	**0.095**	− 0.204	0.016
*age29_39 (Q2)*	0.028	0.091	0.310	0.759	− 0.150	0.206
*age40_50 (Q2)*	0.271***	0.100	**2.710**	**0.007**	0.075	0.467
*age50plus (Q2)*	0.103	0.101	1.010	0.310	− 0.096	0.301
*University (Q3)*	0.077	0.052	1.490	0.137	− 0.024	0.178
*non_single (Q4)*	− 0.041	0.083	− 0.500	0.618	− 0.203	0.121
*non_urban (Q5)*	0.114	0.082	1.390	0.166	− 0.047	0.275
*Working (Q7)*	0.054	0.061	0.900	0.370	− 0.064	0.173
*Environmental Damages (Q15)*	− 0.005	0.044	− 0.120	0.906	− 0.091	0.080
*_cons*	0.092	0.147	0.620	0.532	− 0.196	0.379
*Commerce (Eq. ***3****)**						
*qual_life (qual_life)*	0.256***	0.067	**3.830**	**0.000**	0.125	0.388
*Environment (environment)*	− 0.014	0.040	− 0.350	0.730	− 0.093	0.065
*Female (Q1)*	0.030	0.035	0.840	0.399	− 0.040	0.099
*age29_39 (Q2)*	− 0.050	0.043	− 1.180	0.239	− 0.134	0.033
*age40_50 (Q2)*	0.007	0.070	0.110	0.916	− 0.129	0.144
*age50plus (Q2)*	− 0.018	0.081	− 0.220	0.827	− 0.176	0.141
*University (Q3)*	0.039	0.026	1.460	0.143	− 0.013	0.090
*non_single (Q4)*	− 0.039	0.059	-0.660	0.507	-0.154	0.076
*non_urban (Q5)*	0.022	0.048	0.460	0.648	-0.072	0.116
*Working (Q7)*	0.042	0.030	1.400	0.162	-0.017	0.101
*Financial Damages (Q15)*	0.050*	0.029	**1.740**	**0.083**	-0.006	0.107
*New jobs (Q13)*	0.025	0.039	0.640	0.521	− 0.052	0.102
*Landprice (Q16)*	0.130*	0.074	**1.770**	**0.077**	− 0.014	0.275
*Landuse (Q17)*	0.011	0.027	0.410	0.684	− 0.042	0.063
*Hotels (Q19)*	0.033	0.033	1.000	0.319	− 0.032	0.098
*Restaurants (Q19)*	− 0.075**	0.032	**− 2.380**	**0.017**	− 0.137	− 0.013
*Coffee shops (Q19)*	0.123**	0.055	**2.240**	**0.025**	0.015	0.230
*Commercial shops (Q19)*	0.088	0.069	1.280	0.201	− 0.047	0.222
*Museums (Q19)*	0.010	0.045	0.210	0.831	− 0.079	0.098
*Cultural / Historical sites (Q19)*	0.026	0.047	0.570	0.572	− 0.065	0.118
*Industries (Q19)*	− 0.008	0.037	− 0.230	0.819	− 0.081	0.064
*Agriculture (Q19)*	0.025	0.042	0.600	0.550	− 0.058	0.108
*Industrial District (Q18)*	0.060**	0.025	**2.350**	**0.019**	0.010	0.109
*Urban Infrastructures (Q18)*	− 0.015	0.036	− 0.420	0.674	− 0.085	0.055
*Public Utilities Infrastructures (Q18)*	0.036	0.032	1.120	0.265	− 0.027	0.099
*Open spaces—Urban & Suburban green (Q18)*	− 0.023	0.032	− 0.730	0.463	− 0.086	0.039
*Merchandise Centre (Q18)*	− 0.018	0.025	− 0.730	0.465	− 0.068	0.031
*Holiday Homes (Q18)*	0.038	0.037	1.030	0.304	− 0.034	0.109
*Permanent Residences (Q18)*	0.053*	0.031	**1.750**	**0.080**	− 0.006	0.113
*Urban centres (Q18)*	− 0.009	0.037	− 0.230	0.816	− 0.082	0.064
*Tourist facilities (Q18)*	0.012	0.029	0.410	0.680	− 0.045	0.068
*Wholesale Trade (Q18)*	0.013	0.035	0.360	0.717	− 0.056	0.081
_cons	0.320**	0.136	**2.350**	**0.019**	0.053	0.586
*area_growth (Eq. **4**)*						
*Environment (Model Eq. **1**)*	0.043	0.040	1.080	0.280	− 0.035	0.121
*qual_life (Model Eq. **2**)*	0.213**	0.087	**2.430**	**0.015**	0.041	0.384
*Commerce (Model Eq. **3**)*	0.010	0.066	0.170	0.867	− 0.118	0.140
*Female (Q1)*	0.009	0.037	0.230	0.815	− 0.064	0.081
*age29_39 (Q2)*	0.025	0.051	0.490	0.625	− 0.075	0.125
*age40_50 (Q2)*	0.066***	0.062	**1.060**	**0.291**	− 0.056	0.187
*age50plus (Q2)*	0.192	0.065	2.930	0.003	0.064	0.320
*University (Q3)*	− 0.018	0.041	− 0.450	0.655	− 0.098	0.062
*non_single (Q4)*	− 0.164***	0.060	**− 2.730**	**0.006**	− 0.283	− 0.046
*non_urban (Q5)*	0.017	0.069	0.250	0.805	− 0.118	0.153
*Working (Q7)*	− 0.016	0.042	− 0.380	0.707	− 0.099	0.067
*Financial Damages (Q15)*	− 0.048	0.047	− 1.010	0.314	− 0.141	0.045
*Environmental Damages (Q15)*	0.033	0.032	1.010	0.314	− 0.031	0.096
*Social Damages (Q15)*	− 0.007	0.049	− 0.150	0.883	− 0.104	0.089
*_cons*	0.714***	0.105	**6.790**	**0.000**	0.508	0.920

Highlighted results that are significant in the analysis and discussion indicated in bold

Asterisks *, ** and *** denote statistical significance at 10%, 5% and 1%

As a result, at the correlation control in Table 5, we observe here that the quality-of-life variable (Qual_life) that represents the pillar of Society mutually reinforces all the other pillars that we set, namely the environment (environment), Economy (commerce) and Sustainability (area_growth).

Furthermore, we conducted a Factor analysis with the results in Tables 8, 9, 10 and 11, which provides us with a powerful data reduction technique and enables us to examine the concept of the pillars of sustainable development that cannot be easily measured directly. In addition, factor analysis optimised our model by compressing a great number of variables into a smaller number of underlying factors. This allowed our model to create an efficient pattern for complex data that could be put into action. As can we observe, factor 1 Eigenvalue (Table 8) is more significant than the other two factors and includes the three fundamental pillars of sustainable development; the Uniqueness (Table 10) of the factor’s variables are significant, allowing us to determine that the three pillars are unidimensional. In addition, Table 11 reveals a significant statistical significance at a 10% correlation between factor 1 and the area growth equation representing Sustainable development in our model.

**Table 8:** Factor analysis

Factor	Eigenvalue	Difference	Proportion	Cumulative
Factor1	1.39869	0.50202	0.4662	0.4662
Factor2	0.89666	0.19201	0.2989	0.7651
Factor3	0.70465		0.2349	1.0000
Factor analysis/correlation Number of obs = 266				
Method: principal− component factors Retained factors = 1				
Rotation: (unrotated) Number of params = 3

**Table 9:** Factor analysis correlation

Factor	Variance	Difference	Proportion	Cumulative
Factor1	1.39869		0.4662	0.4662
LR test: independent vs. saturated: chi2(3) = 32.65 Prob > chi2 = 0.0000				
Factor analysis/correlation Number of obs = 266				
Method: principal-component factors Retained factors = 1				
Rotation: orthogonal varimax (Kaiser off) Number of params = 3

**Table 10:** Factor Scoring coefficients

Variable	Factor1
environment	0.47192
qual_life	0.55109
commerce	0.43422
method = regression; based on varimax rotated factors	

*Key finding: The three pillars of growth are unidimensional*

**Table 11:** Correlation between the factor variable and Sustainable Development Equation

	Factor1	Area_growth
Factor1	1.0000	
Area_growth	0.1988*	1.0000

Note: Asterisks * 0.01

User satisfaction was another aspect of research that we want to focus on and attempt to represent by the Eq. (5), which is estimated as a linear probability model. The results are presented in Table 12, with heteroskedasticity robust standard errors in the third column of the table. The obvious result of this regression is that the people who found the prices of use of the infrastructure project are more likely to state that they are satisfied by the mega infrastructure project of Athens Metro. Moreover, the respondents that stated that the project was maintained satisfactorily are more likely to reply that they are satisfied with this infrastructure project (Table 12).

**Table 12:** User satisfaction linear regression analysis

Robust						
	Coef	Std.Err	t	P >|t|	[95% Conf.Interval]	
*area_growth*	0.026	0.060	0.430	0.664	− 0.093	0.145
*female*	0.039	0.045	0.880	0.380	− 0.049	0.127
*age29_39*	0.101	0.062	1.640	0.102	− 0.020	0.223
*age40_50*	0.189***	0.065	**2.890**	**0.004**	0.060	0.318
*age50plus*	0.115	0.072	1.590	0.114	− 0.028	0.258
*University*	− 0.015	0.039	− 0.390	0.694	− 0.093	0.062
*non_single*	-0.045	0.054	-0.820	0.414	-0.152	0.063
*non_urban*	0.004	0.075	0.060	0.955	-0.143	0.151
*working*	-0.007	0.048	-0.150	0.881	-0.101	0.087
*price_reasonable*	0.125***	0.046	**2.710**	**0.007**	0.034	0.215
*mistakes*	-0.122**	0.050	**-2.460**	**0.014**	-0.220	-0.024
*serviced*	0.320***	0.055	**5.860**	**0.000**	0.212	0.427
*whatmore_design*	-0.050	0.061	-0.810	0.419	-0.171	0.071
*whatmore_space*	-0.082	0.053	-1.550	0.122	-0.186	0.022
*_cons*	0.552***	0.096	**5.730**	**0.000**	0.362	0.742
Number of obs = 266						
F (14, 251) = 5.32						
Prob > F = 0.0000						
R-squared = 0.3193						
Root MSE = .3036						

Highlighted results that are significant in the analysis and discussion indicated in bold

Asterisks *, ** and *** denote statistical significance at 10%, 5% and 1%

## Conclusions

The above statistical research analysis findings provide enough valuable information and answers to our primary research objective about the contribution of Athens (Attiko) Metro to Athens' sustainable development through the social lens of understanding the sustainability concept. Furthermore, the research provided clear answers to the Athens Metro user satisfaction question, which is positive, as evidenced by the overall positive results of the analysis of the prototype statistical model of the Sustainable development Pillars and the Users Satisfaction equation.

The study offered robust results to the questionnaire's selected questions (Table 4) that were employed in the statistical analysis model. First, the overwhelming majority believe that the areas covered by the project have been developed and have benefited in general due to the Metro’s construction and existence, responding positively to the sustainability pillar. Additionally, the research found that most respondents believed the Athens Metro contributed to the expansion of commerce in the surrounding districts by responding to the economy’s pillar.

Simultaneously, respondents expressed strong views on whether residents’ quality of life in the surrounding areas, which serves as the research’s pillar of the Society, has improved. Therefore, the Metro’s contribution is characterised as beneficial in terms of the surrounding areas’ environmental impact, reflected by the pillar of the environment in the statistical model. Once again, the research indicated very high percentages of acceptance by respondents. The presence of the Metro in the surrounding communities has alleviated many of the previously mentioned issues. Many of these locations have become some of the most popular locations for installation. In addition, the Metro has improved people’s living conditions due to the increased flow of passengers that it transports daily, remodelling the surrounding areas.

In conclusion, we believe that the current research on the contribution of large infrastructure projects to sustainable development requires a great deal of analysis. Therefore, the aim is to continue further investigation of the statistical model, enriching it with secondary statistics of statistical services and organisations, adding independent and dependent formulas of the fundamental pillars of Sustainable Development. The ultimate goal is to create a reliable statistical model for analysing the impact of large infrastructure projects on sustainable development.

## Footnotes

1 The findings are robust to the choice of estimation technique, and they are confirmed when employing nonlinear estimators, such as a Probit estimator. The estimates are not presented here for space considerations.
